# Predicting Outcome in Clear Aligner Treatment: A Machine Learning Analysis

**DOI:** 10.3390/jcm13133672

**Published:** 2024-06-24

**Authors:** Daniel Wolf, Gasser Farrag, Tabea Flügge, Lan Huong Timm

**Affiliations:** 1 Independent Researcher, Berlin 13089, Germany; dwolfeu@gmail.com; 2 Straumann Group—etkon GmbH, Lochhamer Schlag 6, 82166 Gräfelfing, Germany; gasser.farrag@straumann.com; 3 Department of Oral and Maxillofacial Surgery, Charité—Universitätsmedizin Berlin, Corporate Member of Freie Universität Berlin and Humboldt-Universität zu Berlin, Hindenburgdamm 30, 12203 Berlin, Germany; 4 DrSmile—DZK Deutsche Zahnklinik GmbH, Königsallee 92a, 40212 Düsseldorf, Germany

**Keywords:** orthodontics, machine learning, artificial intelligence, clear aligners, malocclusion, prognosis optimization, prediction

## Abstract

**Background/Objectives:** Machine learning (ML) models predicting the risk of refinement (i.e., a subsequent course of treatment being necessary) in clear aligner therapy (CAT) were developed and evaluated. **Methods:** An anonymized sample of 9942 CAT patients (70.6% females, 29.4% males, age range 18–64 years, median 30.5 years), as provided by DrSmile, a large European CAT provider based in Berlin, Germany, was used. Three different ML methods were employed: (1) logistic regression with L1 regularization, (2) extreme gradient boosting (XGBoost), and (3) support vector classification with a radial basis function kernel. In total, 74 factors were selected as predictors for these methods and are consistent with clinical reasoning. **Results**: On a held-out test set with a true-positive rate of 0.58, the logistic regression model has an area under the ROC curve (AUC) of 0.67, an average precision (AP) of 0.73, and Brier loss of 0.22; the XGBoost model has an AUC of 0.67, an AP of 0.74, and Brier loss of 0.22; and the support vector model has a recall of 0.61 and a precision of 0.64. The logistic regression and XGBoost models identify predictors influencing refinement risk, including patient compliance, interproximal enamel reduction (IPR) and certain planned tooth movements, for example, lingual translation of maxillary incisors being associated with the lowest risk of refinement and rotation of mandibular incisors with the highest risk. **Conclusions:** These findings suggest moderate, well-calibrated predictive accuracy with both regularized logistic regression and XGBoost and underscore the influence the identified factors have on the risk of refinement in CAT, emphasizing their importance in the careful planning of orthodontic treatment and the potential for shorter treatment times, less patient discomfort, and fewer clinic visits. Identification of at-risk individuals could support tailored clinical decision-making and enable targeted interventions

## 1. Introduction

In recent years, clear aligner therapy (CAT) has become increasingly popular as an orthodontic treatment for correcting malocclusion. The range of tooth movements achievable with CAT has expanded [1,2]. However, predicting CAT outcomes, either before or during a course of treatment, and in particular whether a refinement (i.e., a subsequent course of treatment) will be required, is challenging. Accurately predicting the risk of refinement in CAT would be beneficial because it could facilitate better clinical decision-making in treatment planning and addressing complications. From the patient’s perspective, this would mean shorter treatment time, less discomfort, and fewer visits to the dentist or orthodontist, while for the clinician it would mean increased professional satisfaction, since treatment success underlines their expertise and commitment to the patient.

Clinical outcomes are typically predicted by extracting potentially influencing features from medical records, a strategy that requires a priori identification of relevant features. It is known a priori and anecdotally that CAT outcomes depend on patient-related and force-related factors. Moreover, with the increasing use of mobile applications for remote follow-up and teleassistance, factors related to application use have also been found to influence treatment outcomes [3,4]. Nevertheless, the factors that affect CAT outcomes are presently not well understood. The present work is an attempt to improve this understanding.

Currently, no predictive model exists to aid in the improvement of the CAT planning process [5,6]. Although a large number of predictive models have been developed in dentistry to support clinical decision-making, e.g., for periodontal or caries risk assessment or orthodontic extraction patterns [7,8,9,10,11,12], most of them do not consider the full range of available predictors or do not apply to CAT. Machine learning (ML) methods may provide accurate predictions, especially when applied to complex datasets with many variables. This is due to the potential of ML algorithms to capture and reflect the subtleties contained within larger datasets [13,14].

The transparency of a predictive model is crucial in aiding clinical decision-making since an accurate but opaque model only predicts refinement risk and does not indicate how to modify the treatment to reduce this risk. In contrast, a transparent model that identifies both patient-related and force-related factors that affect refinement risk would be a useful tool for clinicians. Based on such a transparent prediction, modifications to the treatment plan and/or follow-up schedule could be made to reduce refinement risk preemptively. Such predictions could be updated as the treatment progresses since refinement may be required mid-treatment [15].

An approach called ‘SHapley Additive exPlanations’ (SHAP) can be used with ML algorithms to provide this transparency [16]. SHAP is an approach in the field of interpretable ML that attempts to develop methods to garner an understanding of an ML model’s predictions. Lack of interpretability is a shortcoming of many so-called ‘black box’ algorithms and problematic in clinical decision-making, where practitioners wish to take preventative action to ameliorate a predicted undesirable outcome, as mentioned above. SHAP is a post hoc, theoretically principled, and local approach that, for each prediction, generates a so-called ‘SHAP value’ for each of the model’s predictors such that these SHAP values sum to the model’s prediction. The sign (positive or negative) and relative size of each SHAP value explain that predictor’s influence on the prediction, thereby providing the transparency practitioners require.

The present study explores the factors influencing the success of planned tooth movements in adult patients undergoing CAT. Longitudinal tooth movement, demographic, and treatment compliance data from electronic medical records are used to develop ML models that predict refinement risk and to evaluate whether these results are consistent with clinical considerations. The findings could provide valuable insights into predicting CAT outcomes for the first time and pave the way for further research.

## 2. Materials and Methods

### 2.1. Study Design

Three different ML approaches were used to predict treatment outcome: (1) logistic regression with L1-regularization (henceforth ‘lasso logistic regression’); (2) XGBoost, an open-source ML algorithm based on gradient boosting; and (3) support vector classification with a radial basis function kernel (henceforth ‘SVC-RBF’). Summaries of these techniques are given in Section S3 of the Supplementary File. The decision to use three different methods, rather than a single method, was made to make the study more robust. The particular methods were chosen for their distinct approaches to prediction: logistic regression is a linear method; XGBoost is based on decision trees; and SVC-RBF uses a non-linear, local similarity function.

Another important objective addressed by the study design is interpretability. Although accurate predictions of treatment outcomes are desirable, they are of limited use for clinical decision-making if nothing can be done to ameliorate a predicted unsuccessful outcome. A logistic regression model is straightforward to interpret using the coefficients of the covariates, but this is not the case for the other two methods selected, XGBoost and SVC-RBF. For this reason, it was decided to use SHAP with these two methods.

### 2.2. Setting, Participants, and Sample Size

The cohort studied included CAT patients who started treatment between 1 June 2021 and 25 January 2022. Patients were selected for inclusion in the study if they had completed treatment with or without refinement, had a malocclusion treated in the anterior and premolar regions, were adults over the age of 18 with permanent dentition, had no local and/or systemic conditions that could affect bone metabolism, had no periodontal disease, and did not require extractions during CAT. Basic periodontal examinations (BPE) [17,18] and CMD screenings [19] were performed to rule out contraindications to CAT, such as CMD or active periodontal disease. All dentists and orthodontists were licensed professionals with a minimum of three years of experience in using clear aligners. Additionally, they underwent initial and continuous training through the Clear Aligner Academy program. This program ensures that all practitioners are equipped to handle a range of cases, from simple to moderately complex clear aligner therapy (CAT), and to conduct standardized clinical examinations for appropriate case selection within the treatment scope [20]. The treatment protocol employed a series of custom-designed aligners tailored to the complexity of each case. These aligners were characterized by a smooth, unscalloped, high trimline that extended 2 mm above the gingival margin. They were fabricated using ClearQuartz™ Multilayer-Material. This material combines two durable outer layers with a flexible elastomeric core. The hard outer layers enhance durability and stain resistance, while the flexible core minimizes initial pressure and provides sustained force. Each aligner facilitated tooth movements with translation velocities of 0.2–0.3 mm per aligner and rotational movements of 2–3 degrees per aligner. The aligners were designed to be worn for a two-week period, ensuring gradual and consistent tooth alignment. Patients were instructed to wear the aligners for at least 22 h per day, with removal permitted only during meals, hot drink consumption, and oral hygiene routines. This regimen was crucial for maintaining the efficacy and predictability of the treatment outcomes. Of the 9983 patients in the sample, 41 were removed as part of data cleaning (see Section S2 of the Supplementary File).

The investigated cohort of 9942 patients (74.4% females, 25.6% males, with an age range of 18–64 years, median 30.5 years) was evaluated retrospectively and without intervention using real-world data provided by DrSmile, a Berlin-headquartered health tech company providing CAT. Patient data were collected as part of routine care and anonymized for research purposes, which, according to the Berlin State Hospital Act (Landeskrankenhausgesetz Berlin) and the recommendations of the Datenschutz und IT-Sicherheit im Gesundheitswesen (DIG) task force of the German Association for Medical Informatics, Biometry, and Epidemiology (GMDS), do not require approval by an ethics committee or informed consent.

The study was conducted in accordance with the World Medical Association Declaration of Helsinki, and the reporting followed the RECORD and TRIPOD Statement [21,22].

### 2.3. Variables

The outcome to be predicted was whether a CAT patient completed their treatment with or without refinement. The predictors were: age (at the start of treatment), gender (self-reported), treatment start date, whether IPR was planned, whether attachments were used, whether the patient checked in their aligners (a measure of treatment compliance), number of treatment steps, and planned tooth movements. Further details are given in Section S2 of the Supplementary File.

### 2.4. Bias

Although including patients from only one CAT provider carries a risk of selection bias (see Section 3.1), we ensured that our sample included all patients who underwent CAT within a given timeframe to ensure a comprehensive sample, except for a small number of patients (41 out of 9983) who were removed from the data as outliers (see Section S2 of the Supplementary File).

## 3. Results

### 3.1. Descriptive Statistics

A total of 7021 (70.6%) patients in the sample are female and 2921 (29.4%) male. The median age is 30.5 years (with a range of 18 to 64 years). By age group, older adults are the smallest group (older than 55 years, n = 190, 1.9%), followed by young adults (18–24 years, n = 2211, 22.2%) and middle-aged adults (35–54 years, n = 2788, 28.0%), while adults (25–34 years, n = 4753, 47.8%) are the largest group.

### 3.2. Performance of the Models

**Figure 1:**
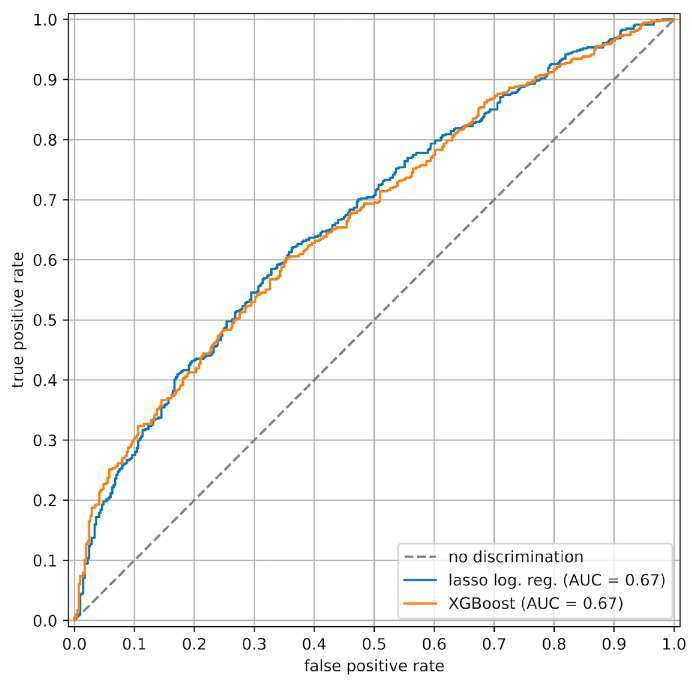
ROC curves of the lasso logistic regression and XGBoost models.

**Figure 2:**
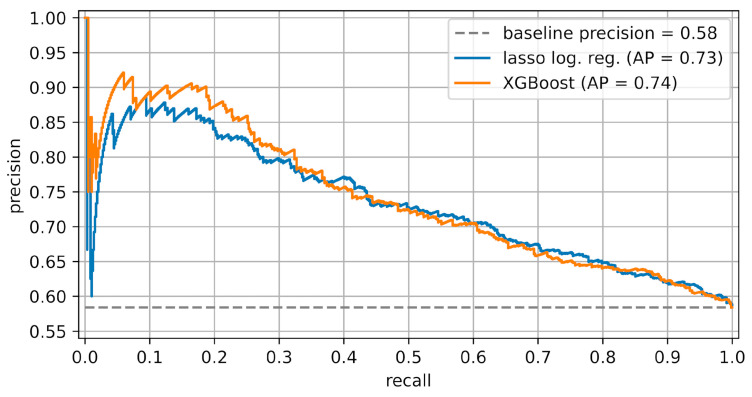
Precision–recall curves of the lasso logistic regression and XGBoost models. The baseline precision is the proportion of refinement cases in the hold-out test dataset.

**Figure 3:**
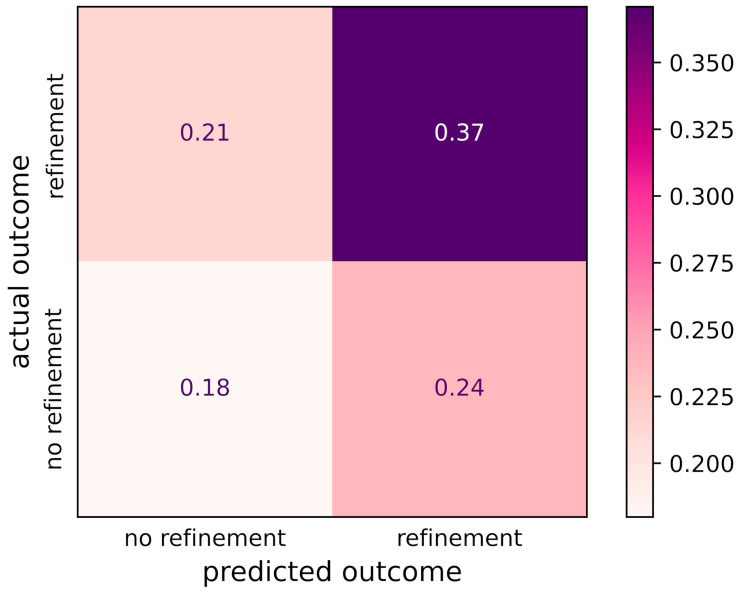
Confusion matrix of the SVC-RBF model.

The models were evaluated on a test dataset that was not used to train the models (see Section S3 of the Supplementary File). The true positive rate (i.e., the proportion of refinements) in this test dataset is 0.58. The lasso logistic regression model has an area under the ROC curve (AUC) of 0.67 and an average precision (AP) of 0.73, while the AUC and AP of the XGBoost model are 0.67 and 0.74, respectively: see Figure 1 and Figure 2. The lasso logistic regression and XGBoost models are well calibrated, each with a Brier loss of 0.22; see Figure S1 of the Supplementary File for calibration curves. The confusion matrix of the SVC-RBF model is shown in Figure 3. Its recall is 0.61 and its precision is 0.64.

### 3.3. Interpretation of the Models

The lasso logistic regression model contains 53 non-zero coefficients. The significant (*p* < 0.05) coefficients are shown in Table 1, along with confidence intervals at the 95% level and a column indicating whether the *p* value is below the Bonferroni threshold, namely 0.05/53 ≈ 0.001. Although not significant (*p* > 0.05), the coefficient of the constant term (the intercept) is also included in the table to indicate the model’s baseline prediction. The scaled coefficients apply to the scaled values of the features (as required for the regularization), while the unscaled coefficients apply to the original unscaled values; see Section S4 of the Supplementary File for further details.

**Figure 4:**
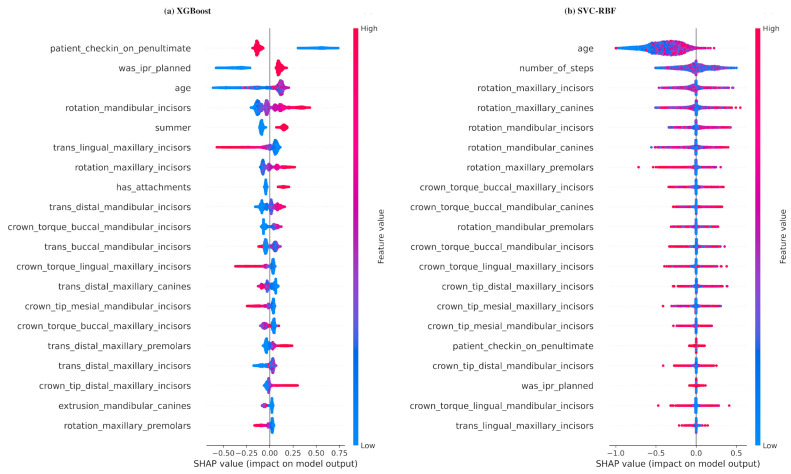
Beeswarm plots of the SHAP values of each model’s predictions for the patients in the training dataset: (**a**) XGBoost (**b**) SVC-RBF.

Figure 4a is a beeswarm plot of the SHAP values of the XGBoost model’s predictions for the patients in the training dataset; see Section S5 of the Supplementary File for a detailed explanation. The predictors are ordered by the mean magnitude of the SHAP value (top to bottom in descending order); only the top 20 predictors under this order are shown. Table 1 and Figure 4a are in broad agreement.

The SVC-RBF model was also interpreted using SHAP. Its beeswarm plot (Figure 4b) shows no discernible patterns. This is consistent with the poor performance of the model (Figure 3).

## 4. Discussion

In the present study, data from 9942 European CAT patients were used to train and test ML models; the underlying dataset contained 74 features. The specific aims of the study were to develop ML models that predict refinement risk and to evaluate whether these results are consistent with clinical considerations.

Chekroud et al. highlighted the potential of machine learning in psychiatry, indicating its capability to sequence treatments over time or design individualized treatment protocols. This concept of personalized and customized treatment has shown benefits in various healthcare areas, including smoking cessation, breast cancer screening, and physical activity [23]. Several recent studies have demonstrated that machine learning, including techniques like support vector machines, outperforms traditional regression methods. Large-scale comparisons using benchmark datasets consistently confirm the superior effectiveness of machine learning [23,24]

Similarly, in clear aligner therapy, machine learning can enhance treatment planning by predicting individual treatment outcomes and identifying the most influential factors.

By leveraging patient-specific data, our predictive models can assist orthodontists in tailoring treatment plans to the unique needs of each patient, potentially improving the efficacy and efficiency of clear aligner therapy.

Our study employed three approaches for predicting CAT treatment outcomes: logistic regression with L1-regularization (lasso logistic regression), XGBoost, and support vector classification with a radial basis function kernel (SVC-RBF). The lasso logistic regression and XGBoost models yielded moderate predictive accuracy, with AUC values of 0.67 (Figure 1) and average precisions of 0.73 and 0.74 (Figure 2), respectively. Moreover, the SHAP values of the XGBoost model (Figure 4a) provide useful interpretations of the model. In contrast, the performance of the SVC-RBF model was poor, with a recall of 0.61 and a precision of 0.64 (see Figure 3 for the confusion matrix), and its SHAP values (Figure 4b) do not lend themselves to easy interpretation: while age, number of steps, and various rotations and crown torque movements are important in the model’s predictions, there are no clear global relationships between the predictor values and their SHAP values, with the possible exception of age, whose SHAP values suggest that lower age is associated with a reduced risk of refinement. We now return to the lasso logistic regression and XGBoost models, discussing various points in detail.

Firstly, an interesting finding of this study is the broad agreement between the logistic regression and XGBoost models, both in terms of their model performance and the importance assigned to various features. This alignment underscores the significance of the models’ performances and the features thereby identified.

Secondly, certain types of tooth movement and other covariates emerge as the most influential predictors of refinement. Lingual translation of maxillary and mandibular incisors, robust patient compliance, mesial crown tipping of mandibular incisors, and younger age are associated with a lower risk of refinement. In contrast, factors such as the rotation of maxillary and mandibular incisors, distal crown tipping of maxillary incisors, planned interproximal reduction (IPR) or the presence of attachments may elevate the risk of refinement. These results are consistent with previous research highlighting less predictability with certain types of tooth movement, such as rotations. For example, Rossini et al. [25] and Charalampakis et al. [26] reported that rotations were more difficult to control, often with smaller rotations achieved than initially planned. Lombardo et al. [27] have identified mesiodistal tilting and vestibulo-lingual tilting as the most predictable movements, with rotations being less predictable. This observation is supported by the research of Bilello et al. [28] and Haouili et al. [29], who highlighted the accuracy of vestibulo-lingual tipping as a movement.

Thirdly, as mentioned above, the presence of IPR was found to be a predictor associated with an increased risk of refinement. This may be related to clinical research findings indicating that the actual amount of enamel removed during IPR procedures in vivo frequently does not correspond with the initially planned IPR amount. Typically, these clinical outcomes tend to result in a lower degree of enamel removal than originally intended values [30].

Fourthly, our study showed the correlation between initiating treatment during the summer months and an increased likelihood of refinement. This observation aligns with previous research that has found reduced compliance during the summer season, potentially due to various distractions associated with favorable weather conditions (Lee 2014; Timm 2022). Furthermore, we found younger age to be associated with a lower refinement risk and, therefore, a shorter treatment duration. This is consistent with many studies that have found age-related effects on orthodontic tooth movement (OTM), with younger age showing faster OTM and older age showing a delayed response to orthodontic forces [31,32].

These findings contribute to a deeper understanding of the complex factors influencing CAT outcomes and highlight the clinical relevance of considering specific tooth movements, patient compliance, age, and seasonal variations when tailoring treatment plans and setting patient expectations. We draw attention to the utility of waterfall plots of SHAP values (Figure S3 in the Supplementary File), which provide a clear overview of the factors influencing an individual prediction, thereby acting as an aid to clinical decision-making.

The strengths of this study include the first use of ML to predict CAT outcomes. This is particularly noteworthy given that research suggests that certain types of tooth movement are more predictable than others. Second, in addition to tooth movement data, a wide variety of other factors were used as predictors, including patient compliance data, some of which may not at first glance be directly related to CAT results, such as the month of treatment start. Complex big data, as part of advanced analytics, can uncover complex and/or hidden patterns in data that are beyond the feasibility of conventional investigation, creating accurate models that were previously inaccessible. As a result, analytics help dentists make informed treatment decisions that can lead to more efficient planning, satisfied patients, and better outcomes. Lastly, it offers the potential to identify at-risk patients and provide them with electronic reminders or schedule more frequent check-ups, thereby mitigating treatment risks and optimizing complex decisions concerning unexpected treatment outcomes and potential refinement risks.

However, the present study has certain limitations. Firstly, although three different ML methods were used in this study, other techniques are also available, e.g., the random forest algorithm or logistic regression with elastic net regularization [33]. Artificial neural networks are also a possibility, although 10,000 data points might prove to be limiting for such a technique [34]. Secondly, adults aged 55 or above were underrepresented in the data. Thirdly, the models were developed using data from a single provider, which may limit their generalizability. Further studies, such as a prospective clinical trial, are needed, given the retrospective design of the current study. Other potential avenues of investigation include further feature engineering [35], deeper analysis of the SHAP values obtained, and causal analysis of the models.

Regardless of these shortcomings, the current study shows promise in aiding orthodontic practitioners to assess refinement risk and thereby tailor treatment plans accordingly. Ultimately, improving the predictability of CAT outcomes will benefit both patients and orthodontic professionals, and this study is a step forward in understanding and predicting CAT treatment outcomes, enabling early modification of the treatment planning strategies beyond current knowledge and best practices, and informing future research.

## 5. Conclusions

Orthodontics has increasingly adopted clear aligner therapy (CAT) as a discreet and efficient method for achieving orthodontic treatment goals [1,2]. Yet, predicting treatment outcomes and the risk of refinement in CAT remains challenging. This study aimed to address this gap by developing prediction models using machine learning and real-world data to explore factors influencing the outcome of planned treatments in adult CAT patients. To the authors’ knowledge, this is the first study to use machine learning to predict the risk of refinement in CAT patients. The lasso logistic regression and XGBoost models yielded useful, well-calibrated accuracy in predicting individual refinement risks and consistently identified several factors influencing refinement risk. These promising results suggest that machine learning algorithms can be effectively used to predict treatment outcomes in CAT and support clinical decision-making.

## Figures and Tables

**Table 1:** The scaled and unscaled coefficients of the lasso logistic regression model. The units given in brackets apply to the unscaled values of the covariates. Only those coefficients with a *p* value of less than 0.05 are shown. The 95% confidence intervals of the scaled coefficients and whether the *p* value is below the Bonferroni threshold are given. The rows are ordered by the magnitude of the scaled coefficient. All values in the table are rounded to two decimal places.

Covariate	Scaled Coefficient	Unscaled Coefficient	95% Confidence Interval of Scaled Coefficient	*p* Value < Bonferonni Threshold
lingual translation of maxillary incisors (mm)	–1.27	–0.25	(–1.79, –0.74)	yes
rotation of mandibular incisors (°)	0.97	0.03	(0.62, 1.32)	yes
lingual translation of mandibular incisors (mm)	–0.80	–0.15	(–1.59, –0.01)	no
distal crown tip of maxillary incisors (°)	0.73	0.05	(0.19, 1.27)	no
patient checked in aligners up to and including penultimate check-in (Boolean)	–0.72	–0.72	(–0.83, –0.61)	yes
rotation of maxillary incisors (°)	0.67	0.02	(0.32, 1.03)	yes
mesial crown tip of mandibular incisors (°)	–0.66	–0.05	(–1.27, –0.05)	no
was IPR planned (Boolean)	0.47	0.47	(0.35, 0.58)	yes
age between 18 and 24 (Boolean)	–0.37	–0.37	(–0.49, –0.26)	yes
summer (Boolean)	0.28	0.28	(0.18, 0.39)	yes
has attachments (Boolean)	0.24	0.24	(0.12, 0.36)	yes
male (Boolean)	0.10	0.10	(0.00, 0.20)	no
constant term (logit)	0.09	0.09	(–0.10, 0.29)	*p* > 0.05

## Data Availability

Data available on request due to privacy restrictions.

## Supplementary Materials

The following supporting information can be downloaded at: https://www.mdpi.com/article/10.3390/jcm13133672/s1, Figure S1: Calibration curves and Brier scores of the lasso log. reg. and XGBoost models. The estimated probabilities are grouped into ten bins of equal width: between 0 and 0.1, between 0.1 and 0.2 and so on. Bins containing two or fewer points (see Figure 4) were excluded from the plot.; Figure S2: Distributions of the estimated probabilities of the lasso log. reg. and XGBoost models. The estimated probabilities are grouped into ten equally wide bins: between 0 and 0.1, between 0.1 and 0.2, and so on.; Figure S3: Waterfall plot of the SHAP values of the XGBoost model’s prediction for a patient in the training dataset. The predicted probability of refinement is 0.65 (≈sigmoid(0.202)). Positive SHAP values are shown in red and negative SHAP values in blue. A higher predicted value means a higher refinement risk: red/positive means an increased refinement risk, while blue/negative means a decreased refinement risk. For example, the model considers poor compliance (patient_checkin_on_penultimate = 0) to increase the refinement risk (SHAP value = +0.49), while the absence of planned IPR (was_ipr_planned = 0) has a similarly sized but downward effect on the refinement risk (SHAP value = –0.45) [33,36,37,38,39,40,41].
