# Supplementary material for: Predicting Outcome in Clear Aligner Treatment: A Machine Learning Analysis
Source: J Clin Med. 2024 Jun 24;13(13):3672. doi: 10.3390/jcm13133672 (PMC11242237; doi:10.3390/jcm13133672)
Supplement: Supplementary file 1 [file jcm-13-03672-s001.zip › jcm-3039684-supplementary.pdf]

Supplementary appendix to ‘Predicting outcome in clear aligner treatment: A machine learning analysis’

Daniel Wolf, Gasser Farrag, Tabea Flügge and Lan Huong Timm

1. Software

Data preparation, analysis, model training, model evaluation, and visualization were performed using Python (version 3.10.3). We provide details of the specific Python software libraries used: Data preparation was performed using version 2.0.0 of pandas (https://pandas.pydata.org/), a library for data transformation and analysis. Plots were generated using version 3.4.3 of matplotlib (https://matplotlib.org/), version 0.12.2 of seaborn (https://seaborn.pydata.org/), and version 1.2.2 of scikit-learn (https://scikit-learn.org/). Lasso logistic regression was performed using version 0.13.5 of statsmodels (https://www.statsmodels.org/), a library that provides implementations of numerous statistical methods. XGBoost has been implemented in several programming languages; in this study version 1.7.5 of the Python library was used (https://pypi.org/project/xgboost/). The implementation of support vector classification used was that in version 1.2.2 of the aforementioned scikit-learn, a library containing implementations of numerical machine learning methods. SHAP calculations were performed using version 0.41.0 of its implementation in Python (https://pypi.org/project/shap/).

2. Data preparation

The data used in this study were extracted in January 2023. Demographic, IPR, and compliance data for the first 10,000 patients (ordered by treatment start date) who started treatment on or after 1 June 2021 were extracted. The last patient in this group started treatment on 25 January 2022. For the same period, data on planned tooth movements and attachments were extracted. This dataset included 9999 patients. The two datasets were joined using a common key. The resulting joined dataset contained data from 9983 patients; the reason for this discrepancy was not identified but was likely due to small differences in how the data were extracted from the two systems. Seven patients were removed from the dataset because their age in the dataset was less than 18 years. A total of 25 patients aged 65 years and over were removed, as they presented a small-sized age group relative to the size of the cohort. Patients with a very low and a very high number of treatment steps were also removed, namely, four patients whose treatment consisted of only five aligners and five patients whose treatment consisted of 22 aligners. The resulting dataset comprised 9,942 patients.

Binary variables were encoded as boolean variables. For instance, gender = female was encoded as 0 and gender = male as 1. (Female was taken as the baseline because the dataset contained more female patients than male patients: see section 3.1 of the main article.) The outcome variable was also encoded as a boolean variable: 0 for no refinement, 1 for refinement. (In keeping with standard terminology, cases of no refinement are sometimes referred to as 'negative cases' and cases of refinement as 'positive cases', even though no refinement – i.e. a successful course of treatment – is a positive outcome for the patient in the everyday sense of the word.)

For the XGBoost and SVC-RBF models, age was left as a continuous variable because these models can detect non-linear relationships. For the lasso logistic regression model, which is linear, age was discretized into age ranges 18–24, 25–34, 35–44, 45–54, and 55–65. These age groups were in turn encoded using one-hot encoding. The column encoding the most populous group (25–34) was removed in order to avoid multicollinearity.

The time of year was encoded by adding two new derived features: summer and winter. Patients who commenced treatment in June, July, or August 2021 were assigned the value 1 for the feature summer, while patients who commenced treatment in December 2021 or January 2022 were assigned the value 1 for the value winter.

In the context of CAT, a patient’s treatment compliance can be gauged by whether they check in their aligners. This pattern suggests that they adhere to the prescribed treatment regimen, which involves changing their aligners within the recommended wearing time and wearing them adequately. By changing the aligners in time, patients also receive electronic reminders of their treatment appointments, e.g. for interproximal enamel reduction (IPR)

or attachments. Accordingly, the feature selected to measure compliance was whether patients checked in all aligners in the mobile application up to and including the penultimate check-in. This approach disregarded the final check-in because even compliant patients may opt to skip it, as it occurs after the completion of their treatment. Other indicators such as app usage or photo submissions were available but not used to avoid complexity.

Planned tooth movements were grouped by tooth type and tooth movement, with the aggregated value of each group taken as the mean movement within each group. The tooth types are incisor, canine, premolar and molar. The tooth movements are extrusion, intrusion, buccal translation, lingual translation, mesial translation, distal translation, distal crown-tip, mesial crown-tip, lingual crown-torque, buccal crown-torque, and rotation, where rotation was considered symmetrically. This resulted in 88 tooth-movement features (= 4 types of tooth $\times$ 2 maxillary/mandibular $\times$ 11 types of movements). The decision to group the planned tooth movements in this way was made in order to reduce the number of tooth-movement features (from 352 to 88), thereby making both model training and any subsequent interpretations of the models easier. The grouping is justified clinically since the teeth were grouped into anatomically meaningful groups. It also reduced the variation inflation factors (VIF): After removing constant and near-constant features (see the next paragraph), the largest VIF among the ungrouped tooth movements was 7.2, while among the grouped tooth movements it was 4.0.

Any feature where more than 99% of the values were the same was removed, resulting in the removal of 22 features. This occurred when a particular tooth movement was never or rarely planned, such as the extrusion of maxillary molars.

After all feature selection and processing, the dataset contained 74 features. The dataset used for the lasso logistic regression model contained 77 features since the age feature was discretized, as explained earlier in this section.

3. Machine learning models, training and evaluation

Lasso logistic regression is logistic regression with L1-regularization, a type of regularization also known as (the) lasso (Tibshirani 2018). L1-regularization penalizes large coefficients, to the extent that it can force coefficients to zero (unlike L2-regularization, also known as ridge regression), thereby acting as a method of feature selection.

XGBoost is an ML algorithm based on gradient boosting of decision trees (Krishnapuram et al. 2016). Boosting is a technique that iteratively combines a sequence of weak learners, typically (shallow) decision trees, into a single strong learner. Gradient boosting is a generalization of this technique that uses gradient descent, a versatile method from mathematical optimization.

SVC-RBF is a specific case of a class of machine learning classifiers that attempt to find boundary-separating outcomes in a transformed feature space via the 'kernel trick', whereby a computationally tractable function (the kernel) implicitly describes the geometry of the higher dimensional transformed feature space (Haussler et al. 1992). This allows linear methods to be used in the transformed space without the need for explicit coordinate calculations in the transformed space. In the case of SVC-RBF, the kernel used is the radial basis function, a non-linear, local similarity function between two points.

There were no missing values in the dataset. The dataset was randomly split into two datasets: a training dataset of 8947 points and a test (or validation) dataset of 995 points. Only the training dataset was used to train the models, while the test dataset was exclusively used to evaluate the models after their training was completed.

Hyperparameters are parameters of a learning algorithm that need to be set before any training can take place. Using lasso logistic regression as an example: The regularization parameter is a hyperparameter whose value must be chosen before fitting the model to any data, while the coefficients of the covariates are model parameters determined by the method used to fit the logistic model to the data, e.g. maximum likelihood. Hyperparameter selection for the three methods used was performed using an iterative grid search and 5-fold cross-validation (CV) on the training dataset; see James et al. for an exposition of these techniques (James et al. 2013). The five folds were selected randomly and then used consistently for all the methods. Once the values of the hyperparameters

for a given model had been selected, the model was retrained on the complete training dataset and then its performance was evaluated on the held-out test dataset.

As the lasso logistic regression and SVC-RBF models use feature regularization, all features were scaled to the interval [0, 1] using min-max scaling. The scaling was determined only by the training dataset (or training folds in the case of cross-validation) to avoid data leakage: Each feature was scaled based on the minimum and maximum values contained in the training dataset (training folds). Scaling was not necessary for the XGBoost model because its decision rules use cutoff values.

We explain the scoring methods used for cross-validation. Lasso logistic regression and XGBoost produce predictions as probabilities and so the log loss was used to score performance on the test folds. SVC RBF is a classifier and outputs either 0 (no refinement) or 1 (refinement), so the log loss cannot be used (because the logarithm of zero is undefined); the balanced accuracy was used instead to score performance on the test folds. The balanced accuracy is the arithmetic mean of the sensitivity (= true positive rate) and the specificity (= true negative rate).

We now explain how each model was evaluated on the held-out test dataset. For the lasso logistic regression and XGBoost models, receiver operating characteristic (ROC) curves were plotted and the area under each curve (AUC) was calculated. Precision–recall curves were also plotted and the area under each curve – the average precision (AP) – was calculated. As a binary classifier, ROC and precision-recall curves are not appropriate for SVC-RBF. For this model, the confusion matrix was calculated, along with the precision and the recall.

ROC curves and the AUC are standard ways of assessing a model's ability to discriminate, i.e. how well the model can distinguish negative from positive cases. In fact, the AUC has an intuitive probabilistic interpretation: The AUC is the probability that, given a randomly selected negative point (no refinement) and a randomly selected positive point (refinement), the negative point has a lower estimated probability than the positive point.

An often overlooked but important aspect of model performance is calibration (Calster et al. 2019). Lasso logistic regression and XGBoost produce predictions as probabilities, but are these estimated probabilities meaningful measures of risk? In statistical parlance: Are the models well-calibrated? For instance, if a model estimates a 25% chance of refinement for each of eight patients, would you expect two of them to return to your clinic for refinement? To illustrate the distinction between discrimination and calibration, consider the following hypothetical model: It estimates a "probability" of 0.98 for each case of no refinement and 0.99 for each case of refinement. This model has perfect discrimination – its AUC is equal to 1 – but its calibration is extremely bad: If the model estimates 0.98 for a patient, then the patient does *not* have a 2% chance of refinement. To this end, calibration curves (also known as reliability diagrams) were plotted and the Brier loss (= the mean squared error of the model's predictions) was calculated. Figure 1 in this appendix shows the calibration curves (aka reliability diagrams) of the lasso logistic regression and XGBoost models based on discretizing the estimated probabilities into ten bins of equal width: between 0 and 0.1, between 0.1 and 0.2, and so on. appendix Figure 2 shows the distribution of the estimated probabilities under this discretization.

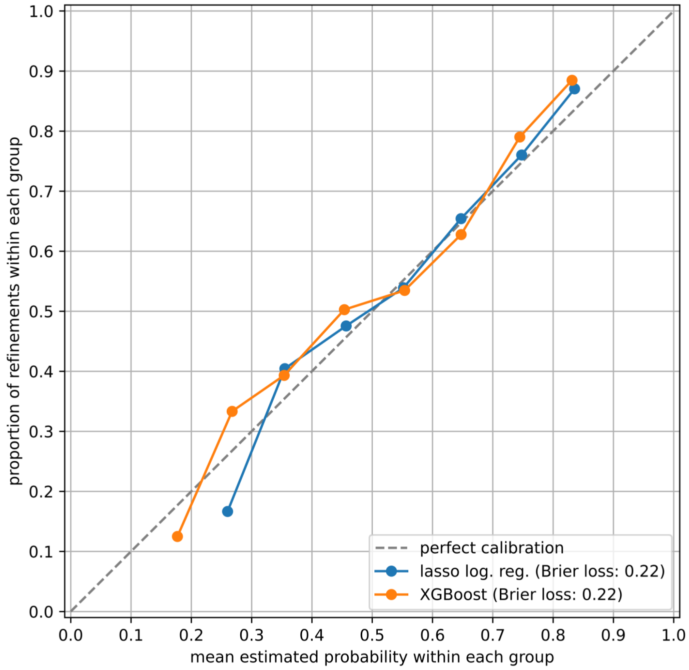

Figure S1. Calibration curves and Brier scores of the lasso log. reg. and XGBoost models. The estimated probabilities are grouped into ten bins of equal width: between 0 and 0.1, between 0.1 and 0.2 and so on. Bins containing two or fewer points (see Figure 4) were excluded from the plot.

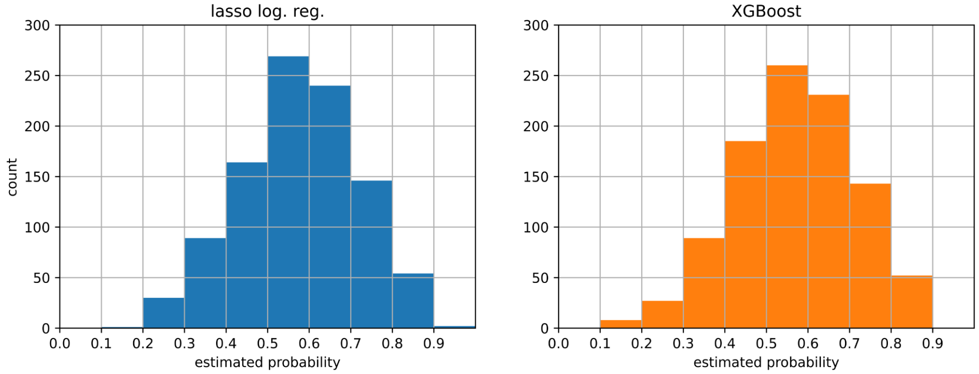

Figure S2. Distributions of the estimated probabilities of the lasso log. reg. and XGBoost models. The estimated probabilities are grouped into ten equally wide bins: between 0 and 0.1, between 0.1 and 0.2, and so on.

4. A detailed explanation of the lasso logistic regression model

The difference between the scaled and unscaled coefficients given in Table 1 in the main article is as follows: As explained in section 3 of this appendix, it is necessary to scale the values of the covariates before training the

model. The scaled coefficients apply to these scaled values. The scaled coefficients are easier to compare with each other because they all lie on the same scale. The unscaled coefficients apply to the original, unscaled values of the covariates. For any readers whose suspicions may be aroused: With the exception of the number of treatment steps, whose coefficient is not significant ($p > 0.05$), each feature's minimum value is zero and thus the scaling and the regression coefficient can be combined into a single multiplicative factor, rather than into a multiplicative part and an additive part shifting the minimum to zero.

The coefficients in Table 1 are for a *logistic* regression model and thus should be interpreted in terms of the odds ratio: Given probability p of refinement, the odds of refinement is the ratio $p / (1 – p)$, i.e. how much more likely refinement is than no refinement. For a covariate x with unscaled coefficient β, $\exp(\beta \cdot x)$ is the odds ratio, i.e. the multiplicative change in the odds given that all other covariates remain the same. For example: (1) The coefficient of the attachment covariate is 0.24. (As a boolean covariate, the scaled and unscaled coefficients are equal.) Thus, according to the model, having attachments increases the odds of refinement by a factor of approximately 1.27 ($\approx \exp(0.24 \cdot 1)$). (2) The unscaled coefficient of lingual translation of maxillary incisors is –0.25. Thus, according to the model, an increase in the mean lingual translation of maxillary incisors by 0.5 mm decreases the odds of refinement by a factor of 0.88 ($\approx \exp(–0.25 \cdot 0.5)$).

Two further remarks concerning the previous paragraph are made. Firstly, the change in the odds ratio is expressed in relative terms. For example, it is assumed that a 0.6 probability of refinement for a given set of covariate values is predicted by the model, where the attachment covariate value is 0. The odds are 1.5 (= 0.6 / 0.4). If the value of the attachment covariate is changed to 1 but the values of all other covariates are left unchanged, then the odds increase to 1.90 (≈ 1.27 · 1.5). However, if the initial predicted probability were 0.2, then the initial odds would be 0.25 (= 0.2 / 0.8) and updated odds 0.32 (≈ 1.27 · 0.25). Secondly, any attempt to interpret the coefficients causatively must be done with great caution. Whilst an increased risk of refinement due to the presence of attachments seems plausible and intuitive, a decreased risk of refinement due to lingual translation of maxillary incisors is harder to interpret. The value of the coefficient is negative and so, according to the model, the more maxillary incisors are lingually translated, the lower the risk of refinement. But do you really believe that translating the teeth more causes the risk of refinement to decrease? When a model is fitted to data, it is confined by its structure and its hyperparameters. In this case, lingual translation of maxillary incisors is negatively correlated with refinement (the Pearson correlation coefficient in the training data is –0.10), i.e. a lot of treatments that involved lingual translation of maxillary incisors were successful. (Bear in mind that the response variable is boolean: 0 for no refinement, 1 for refinement.) The logistic regression model "interpreted" this the only way it can, namely by assigning a negative coefficient to the covariate. A much safer interpretation of this negative coefficient is that the lingual translation of maxillary incisors is easier to achieve than some other movements, even when the lingual translation is large.

5. A detailed explanation of the application of SHAP to the XGBoost and SVC-RBF models

SHAP was used to interpret the XGBoost model, specifically the TreeSHAP (aka TreeExplainer) algorithm (with tree-path dependency), a fast algorithm for calculating SHAP values for tree-based machine-learning models (Lundberg et al. 2020). SHAP is local in that it provides an explanation for each individual prediction, rather than a global explanation of the whole model (as do the coefficients in linear and logistic regression). For example, appendix Figure 3 shows the SHAP values of the XGBoost model's prediction for a patient randomly selected from the training dataset. The SHAP values are ordered by magnitude (top to bottom in descending order). The values of the predictors are given on the vertical axis: For instance, this particular patient did not check in their aligners up to and including the penultimate check-in (0 = patient_checkin_on_penultimate) and the mean planned rotation of the mandibular incisors was 11.75°. The notation $E[f(X)]$ is the expected value of the model's prediction given no predictors, as calculated by TreeSHAP. Note that SHAP uses functional notation to denote models: f denotes the model, X denotes the predictors as a multivariate random variable and x denotes a given set of values of the predictors (i.e. a patient). In this case, the expected value is 0.267. (This value is the same for all patients.) The SHAP values are then added to this value to get the model's prediction, which in this case is 0.202.

Note that this value has the logit as its unit; the model's predicted probability of refinement for this patient is 0.65 (≈ sigmoid(0.202) = 1 / (1 + exp(–0.202))).

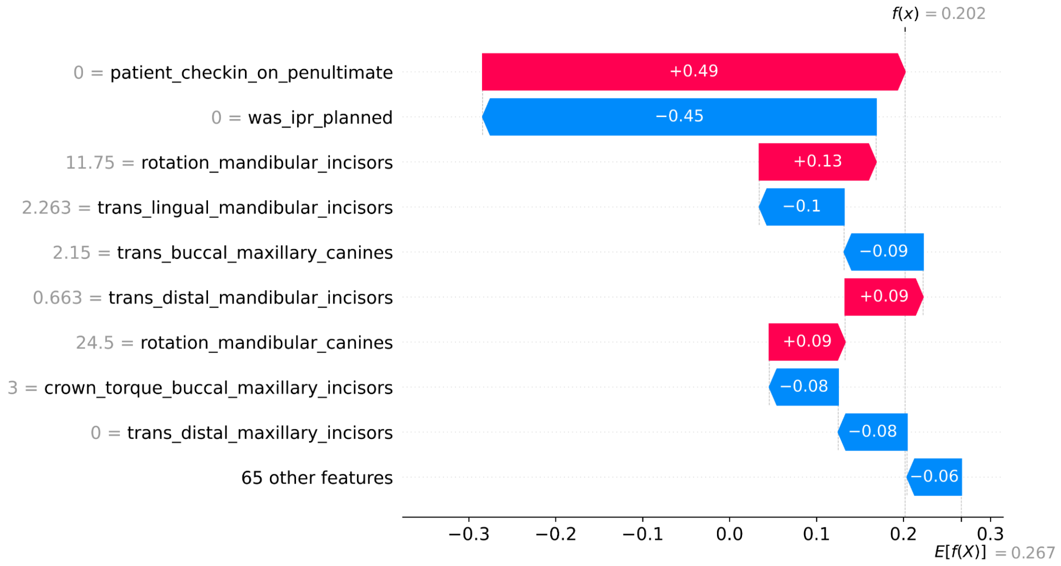

Figure S3. Waterfall plot of the SHAP values of the XGBoost model's prediction for a patient in the training dataset. The predicted probability of refinement is 0.65 (≈ sigmoid(0.202)). Positive SHAP values are shown in red and negative SHAP values in blue. A higher predicted value means a higher refinement risk: red/positive means an increased refinement risk, while blue/negative means a decreased refinement risk. For example, the model considers poor compliance (patient_checkin_on_penultimate = 0) to increase the refinement risk (SHAP value = +0.49), while the absence of planned IPR (was_ipr_planned = 0) has a similarly sized but downward effect on the refinement risk (SHAP value = –0.45).

Whilst inherently local, SHAP values can be collated to give a global interpretation of a model. Figure 4(a) in the main article is a beeswarm plot of the SHAP values of the XGBoost model's predictions for the patients in the training dataset. This is a plot of all SHAP values of all predictions for the given dataset. The predictors are ordered by the mean magnitude of the SHAP value (top to bottom in descending order); only the top 20 predictors under this ordering are shown. In contrast to the waterfall plot (appendix Figure 3), the red/blue coloring now refers to the values of the predictors (not the SHAP values); SHAP values are plotted on the horizontal axis. The beeswarm plot is explained through the following two examples: (1) The predictor patient_checkin_on_penultimate is a proxy for patient compliance: It indicates whether the patient checked in all aligners in the mobile application up to and including the penultimate check-in (see section 2 of this appendix). The variable is binary, so red means a value of 1 (good compliance), and blue a value of 0 (poor compliance). The beeswarm plot shows that values of 1 have negative SHAP values while values of 0 have positive SHAP values. This means that the model associates poor compliance with an increased risk of refinement and good compliance with a decreased risk of refinement. (Recall that the variable to be predicted is also boolean: 0 for no refinement, 1 for refinement.) (2) The predictor rotation_mandibular_incisors are the mean planned rotation among the mandibular incisors (see section 2 of this appendix). Here there is a positive relationship between this predictor and its SHAP values: Larger planned rotations (colored red) have larger positive SHAP values. In other words, the model associates larger planned rotations with a higher risk of refinement.

The SVC-RBF model was also interpreted using SHAP. The KernelSHAP (aka KernelExplainer) algorithm, a model-agnostic approach, was used for the SVC-RBF model. The KernelSHAP algorithm requires a reference dataset: A given predictor is set to its value in this reference dataset and the change in the model's output is

observed. The reference value of each predictor was taken to be zero; this was chosen for conceptual simplicity and because zero is a realistic value for each predictor in the dataset. Figure 4(a) in the main article is a beeswarm plot of the SHAP values of the SVC-RBF model's predictions for the patients in the training dataset. The explanation of the XGBoost's beeswarm plot given in the previous paragraph also applies (*mutatis mutandis*) to Figure 4(b).

A technical note: The calculation of SHAP values involves some theoretical subtleties, primarily concerning the faithfulness of the SHAP values to the distribution of the data and the potential attribution of importance to irrelevant features. Instead of perceiving these aspects as fundamental problems with the SHAP approach, as discussed in Kumar et al. (Kumar et al. 2020), they can be regarded as application-dependent, with some models being true to the data and others true to the model (Chen et al. 2020). In our case, we are more concerned with the real-world relationship between planned tooth movements and the risk of refinement than with the particular machine-learning model itself – we use the latter as a tool to better understand the former. Within this context, the TreeSHAP algorithm falls into the true-to-the-data category, which is consistent with these aims. In contrast, the KernelSHAP algorithm is a true-to-the-model technique. This is unavoidable, since (to the authors' knowledge) it would not be feasible to calculate the observational conditional expectation for a support vector model (Chen et al. 2020). However, the intervention employed, namely taking zero as the reference value for each predictor, is not unrealistic. It is also to be noted that it is the SHAP values of the XGBoost model that have provided useful information.

References

Calster BV, McLernon DJ, Smeden M van, Wynants L, Steyerberg EW, Bossuyt P, Collins GS, Macaskill P, McLernon DJ, Moons KGM, et al. 2019. Calibration: the Achilles heel of predictive analytics. BMC Med. 17(1):230. doi:10.1186/s12916-019-1466-7.

Chen H, Janizek JD, Lundberg S, Lee S-I. 2020. True to the Model or True to the Data? arXiv. doi:10.48550/arxiv.2006.16234.

Haussler D, Boser BE, Guyon IM, Vapnik VN. 1992. A training algorithm for optimal margin classifiers. Proc fifth Annu Work Comput Learn theory.:144–152. doi:10.1145/130385.130401.

James G, Witten D, Hastie T, Tibshirani R. 2013. An Introduction to Statistical Learning, with Applications in R. Springer Texts Stat.:175–201. doi:10.1007/978-1-4614-7138-7_5.

Krishnapuram B, Shah M, Smola A, Aggarwal C, Shen D, Rastogi R, Chen T, Guestrin C. 2016. XGBoost. Proc 22nd ACM SIGKDD Int Conf Knowl Discov Data Min.:785–794. doi:10.1145/2939672.2939785.

Kumar IE, Venkatasubramanian S, Scheidegger C, Friedler S. 2020. Problems with Shapley-value-based explanations as feature importance measures. arXiv. doi:10.48550/arxiv.2002.11097.

Tibshirani R. 2018. Regression Shrinkage and Selection Via the Lasso. J R Stat Soc Ser B: Stat Methodol. 58(1):267–288. doi:10.1111/j.2517-6161.1996.tb02080.x.
